# Levodopa/Benserazide PLGA Microsphere Prevents L-Dopa–Induced Dyskinesia *via* Lower β-Arrestin2 in 6-Hydroxydopamine Parkinson’s Rats

**DOI:** 10.3389/fphar.2019.00660

**Published:** 2019-06-14

**Authors:** Wen-Wen Wang, Xing-Ru Zhang, Jing-Ya Lin, Zeng-Rui Zhang, Zhen Wang, Si-Yan Chen, Cheng-Long Xie

**Affiliations:** ^1^The Center of Traditional Chinese Medicine, The Second Affiliated Hospital, Yuying Children’s Hospital of Wenzhou Medical University, Wenzhou, China; ^2^Department of Neurology, The First Affiliated Hospital of Wenzhou Medical University, Wenzhou, China; ^3^Department of Neurology, Xinhua Hospital, Shanghai Jiao Tong University School of Medicine, Shanghai, China

**Keywords:** β-arrestin2, Levodopa/benserazide PLGA microsphere, Parkinson’s disease, L-dopa–induced dyskinesia, LBPM

## Abstract

Prolonged pulsatile administration of Levodopa (L-dopa) can generate L-dopa–induced dyskinesia (LID). Numerous research has reported that continuous dopamine delivery (CDD) was useful in reducing the severity of LID. 6-OHDA lesioned rats were divided into two groups to receive intermittent L-dopa stimulation (L-dopa/benserazide) or Levodopa/benserazide PLGA microsphere (LBPM) for 3 weeks. rAAV (recombinant adeno-associated virus) vector was used to overexpress and ablation of β-arrestin2. We found that LBPM developed less AIM severity compared with standard L-dopa administration, whereas selective deletion of β-arrestin2 in striatum neurons dramatically enhanced the severity of dyskinesia by LBPM. On the contrary, the effects of LBPM in terms of ALO AIM were further relieved by β-arrestin2 overexpression. Furthermore, no significant change in motor behavior was seen either in inhibition or overexpression of β-arrestin2. In short, our experiments provided evidence that LBPM’s prevention of LID behavior was likely due to β-arrestin2, suggesting that a therapy modulating β-arrestin2 may offer a more efficient anti-dyskinetic method with a low risk of untoward effects.

## Introduction

L-dopa-induced dyskinesia (LID) is a frequent and apparent clinical problem. It is an often disabling motor complication of long-term L-dopa treatment in patients with Parkinson’s disease (PD) (Calabresi et al., 2010). It develops in about 45% of subjects after 5 years and 80% after 10 years of treatment (Rascol et al., 2000; Hauser et al., 2007), which represents a primary therapy limitation and seriously impacts the life quality of LID patients and increases health care burden (Dodel et al., 2001). Until now, the molecular mechanism of LID has remained unclear and its effective administration is limited (Péchevis et al., 2005).

Previous study indicates the pathophysiology of LID mostly likely involves both dopaminergic and non-dopaminergic systems within the basal ganglia (Bargiotas and Konitsiotis, 2013). Although our comprehension of the mechanisms that underlie LID remains incomplete, discontinuous drug delivery of L-dopa is supposed to be involved (Olanow et al., 2006). Under normal physiological conditions in humans, striatal dopamine liberate is relatively steady. Treatment with short plasma-elimination half-life compounds and discontinuous duodenal absorption (L-dopa et al.) is related to swinging plasma levels of the drug and fluctuations in synaptic cleft dopamine concentrations follow (Nutt, 2008). As time passes, this aberrance is supposed to induce maladaptive changes in basal ganglia motor circuits (Morigaki et al., 2017). It has been shown that the pulsatile pattern of L-dopa irritation dopamine receptors (DRs) plays a vital role in the appearance of LID (Aubert et al., 2007). Due to these issues, numerous adjustments to oral L-dopa have been recommended to reduce concentration waves, consisting in a decrease in medication intervals, sustained-release formulations, or combination with catechol-O-methyl transferase inhibitors (COMTI) (Rascol et al., 2011). Although partly efficacious, such strategies cannot realize the effect of continuous dopamine stimulation. Consequently, continuous infusion of soluble L-dopa is an interesting alternative strategy.

Levodopa/benserazide PLGA (Poly Lactic-co-Glycolic Acid) microspheres (LBPM) were employed based on the water-in-oil-in-water (W/O/W) emulsion solvent evaporation method according to our group’s previous publication in order to supply steady plasma concentrations and reduce pulsatile treatment of L-dopa (Yang et al., 2012a; Yang et al., 2012b). The whole course of microencapsulation was completed in a dark environment to avoid the decomposition effect of light on the drugs. Ren et al. found that LBPM acquired persistent release without burst discharge within the first day and had the same release curve from day 2 *in vivo* to day 7 (Ren et al., 2011). We have previously reported that LBPM could be utilized to reduce expression and established LID rats by restraining the expression of DR1/PKA/p-tau as well as PPEB mRNA in dyskintic rats (Xie et al., 2014a). In parallel, our team’s preliminary experiment (data not shown) found that striatal overexpression of β-Arrestin2 alleviated the development of LID by inhibiting D1R signaling. However, whether β-arrestin2 is involved in the mechanisms by which LBPM reduces LID in rats was still unknown. Thus, the present research aimed to investigate the effect of LBPM on β-arrestin2 in LID rats.

## Materials and Methods

### Animals

Fifty-four young male Sprague-Dawley (SD: 4 to 9 months old) rats weighting 200–250 g were used in this experiment (nearly 65% success rate for induction). The animals were housed under controlled lighting conditions (12/12 h cycle), temperature 22 ± 2°C and relative humidity of 55 ± 10%. All the rats had free access to water and food. Animals were acclimated for at least 1 week before the L-dopa/benserazide injections were initiated. All animals were used in compliance with the Institutional Review Board of Wenzhou Medical University and in accordance with the guidelines of the NIH for the care and use of laboratory animals (NIH publication No. 80-23).

### Preparation of LBPM Nanoparticles and Virus Construction

The whole process of preparation of LBPM was performed according to our previous protocol (Xie et al., 2014a). Overexpression or ablation of β-arrestin2 was based on constructing the recombinant adeno-associated virus (AAV) expression vectors (Obio Technology Corp, Shanghai, China). The entire process was based on a previous paper (Urs et al., 2015). Based on the rat brain atlas, the viruses were administered into the nearby ventromedial striatum of unilateral 6-OHDA lesioned rat models *via* a stereotaxic injection as follows: 1) anterior–posterior (AP) +0.9 mm, medial–lateral (ML) −4.5 mm, and dorsal–ventral (DV) −5.0 mm relative to Bregma; as well as 2) AP +0.5 mm, ML −2.5 mm, and DV −4.2 mm. Viruses containing overexpressed/ablated β-arrestin2 vectors groups (β-arrestin2^+/+^ and β-arrestin2^-/-^, respectively) and AAV empty vector groups were infused at a rate of 0.1 μl/min for 10 min (final volume 1.0 μl/site) and the micro-syringe was held in place for an additional 10 min before being slowly withdrawn.

### Induction of L-Dopa-Induced Dyskinesia

PD models were conducted as described before (Wang et al., 2018). Rats were narcotized with 40 mg/kg pentobarbital sodium and were immobilized in a stereotaxic apparatus. The stereotaxic procedure was carried out according to our previous experiment as follows: 6-OHDA hydrochloride (32 μg dissolved in 8 μL) for PD was infused into the right medial forebrain bundle (MFB) with a constant rate of 1 μl/min using the following coordinates (Xie et al., 2014a): 1) AP −3.7 mm, ML −1.7, and DV −7.8; and 2) AP −4.4 mm, ML −1.2 mm, and DV −7.8 mm, and the tooth bar was set to −2.4 mm. The needle was left in place for 10 min before removal. Three weeks after surgery, rats were screened out by the rotations after the use of apomorphine (0.5 mg/kg, i.p.) and the rats displaying more than 7 turns/min toward the opposite side of the lesioned side were included for the following induction of LID: L-dopa and Benserazide (25 mg/kg, 6.25 mg/kg, i.p., respectively) for 3 weeks.

### Drug Treatment

In experiment 1, PD rats in the microsphere groups received LBPM one time/week for 3 weeks and were further divided into three groups: LBPM-L [n = 6, 20 mg/kg, subcutaneous injection (sc)], LBPM-M (n = 6, 40 mg/kg, sc), and LBPM-H (n = 6, 60 mg/kg, sc). L-dopa methyl ester (25 mg/kg), Benserazide (6.25 mg/kg) and 6-OHDA (32 ug dissolved in 8 μl) were acquired from Sigma-Aldrich Co. Ltd (St. Louis, USA) and were freshly prepared in saline containing 0.2% ascorbic acid. Apomorphine hydrochloride (0.5 mg/kg, i.p.) was purchased from Wako Co. Ltd (Japan), dissolved in saline with 0.2% ascorbic acid before use and was administered by intraperitoneal injection in a volume of 2 ml/kg. In experiment two, PD rats received LBPM-M for 1 week, and then AAV-β-arrestin2 (β-arrestin2^+/+^) or AAV-β-arrestin2-shRNA (β-arrestin2^-/-^) were injected into the striatum. Three weeks later, rats were evaluated by the behavioral experiments.

### AIM Ratings

Abnormal involuntary movements (ALO AIMs) were assessed by two members blind to the treatment group at six time points (days 2, 6, 10, 14, 18, and 21 of L-dopa or LBPM administration) as described elsewhere (Xie et al., 2016). For quantiﬁcation of LID, 0 = absent, 1 = present less than 50% of the observation period, 2 = more than 50%, 3 = present each time but stopped by external stimuli and 4 = present each time and was not interfered with by external stimuli. The following three subtypes of ALO AIMs were appraised (Lundblad et al., 2002): axial AIMs, limb AIMs, and orolingual AIMs. The maximum theoretical score per monitoring session was 72.

### Forelimb Functional Test and Apomorphine-Induced Rotation

A quantitative assessment of locomotor activity using forelimb functional test (FFT), modeling clinical symptoms of PD, was performed at four time points (days 3, 8, 13, and 18 of L-dopa or LBPM treatment), was carried out 90 min after L-dopa administrated and was used as an index of parkinsonian disability score. The test was performed as in our previous study (Wang et al., 2018). Moreover, an apomorphine test was also carried out once a week and rotations were quantified for 1 h following injection to evaluate if LBPM affected L-dopa–dependent motor recovery. Experimenters were blind to the FFT and rotation teat.

### Western Blot and Immunofluorescence (IFC)

The process of the Western blot was based on the previous paper (Wang et al., 2018). We targeted the whole striatum for the Western blot. The membranes were blocked with 5% milk in TBS–Tween 20, and then incubated with primary antibodies overnight at 4°C, polyclonal rabbit anti-tyrosine hydroxylase antibody (1:1,000; Millipore) and polyclonal rabbit Anti-Beta Arrestin 2 antibody (1:1,000; Abcam). Then, the membranes were incubated with anti-rabbit horseradish peroxidase IgG (1:1,000; Beyotime Institute of Biotechnology) for 1 h, and the quantification of immunoreactive bands was based on a secondary binding chemiluminescence detection system *via* Quantity One software (Image Lab, Bio-Rad). IFC was according to our previous paper (Xie et al., 2014a). Striatum coronal sections were incubated overnight at 4°C in the primary antibody solution, rabbit anti-Tyrosine Hydroxylase antibody (1:200; Abcam) under fluorescence after 5% BCA in PBS. Scrubbing by PBS three times followed, and slices were hatched away from light in FITC-conjugated goat anti-rabbit IgG (1:1,000, Beyotime Institute of Biotechnology) for 1 h.

### Statistical Analysis

Data were analyzed using Graphed Prism or SPSS 17.0 with correction p values < 0.05 considered significant. All descriptive results are expressed as group means ± standard error of the mean. A two-way ANOVA (time × treatment) was used to analyze behavioral data followed by Bonferroni multiple comparison *post hoc* tests. The data from Western blot or immunohistochemistry conformed to normal distribution and were analyzed using one-way analysis of variance (ANOVA) following LSD *post hoc* comparisons. Area under the curve (AUC) was used to test the effect of LBPM-L, LBPM-M, and LBPM-H on the Global AIM score by the GraphPad Prism. Based on the GraphPad statistical instruction, AUC calculations are equivalent to taking a weighted average of all the Y values, giving the Y values corresponding to the lowest and highest X values half the weight of the other points if the X values are equally spaced (Swift, 1997).

## Results

### LBPM Reduced the Progress of LID Performance

**Figure 1:**
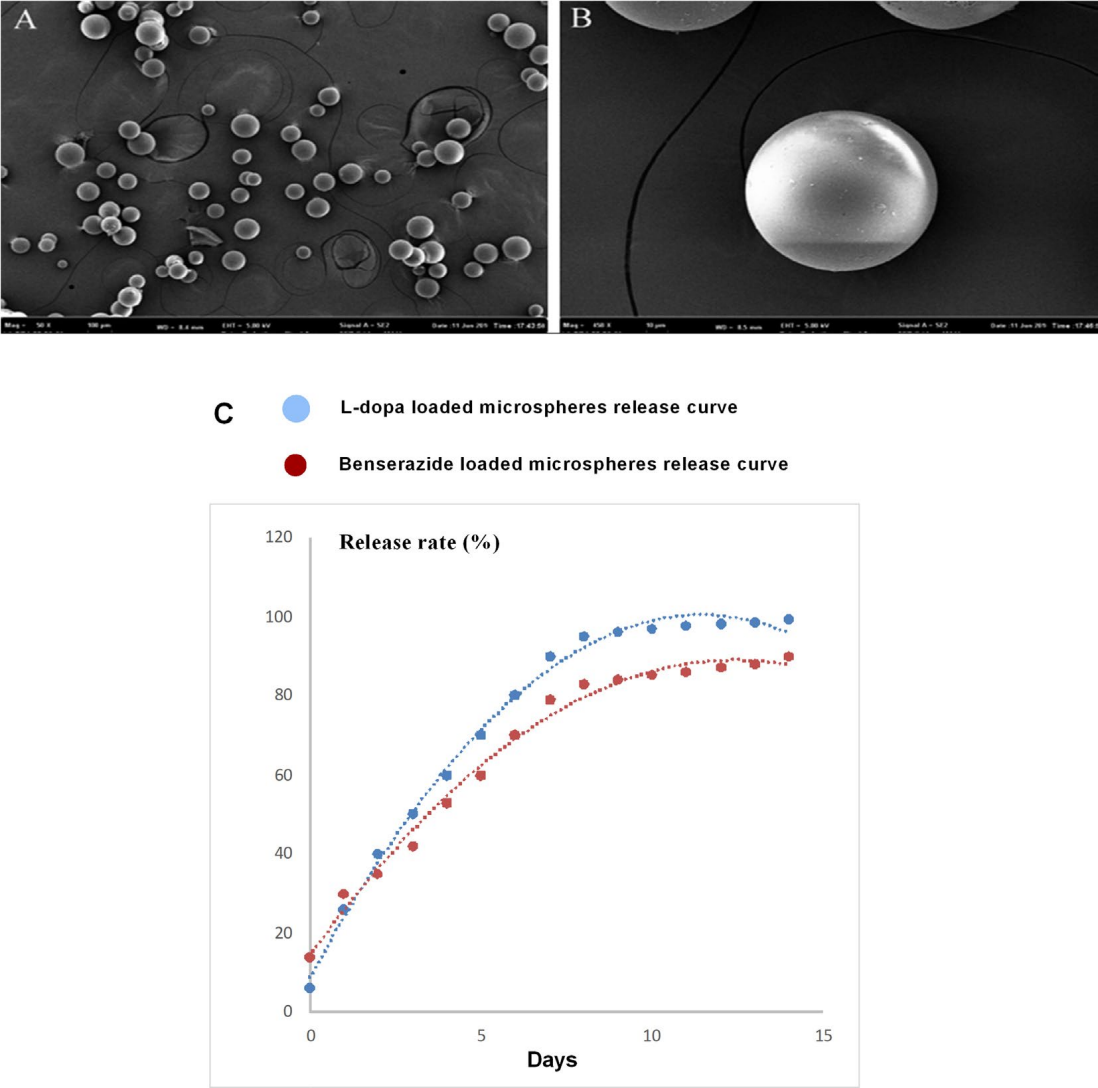
Scanning electron microscopy of the Levodopa/benserazide PLGA microsphere (LBPM) nanoparticles **(A** and **B)**. Release profile of levodopa/benserazide from composite LBPM *in vitro*. n = 4 **(C)**.

**Figure 2:**
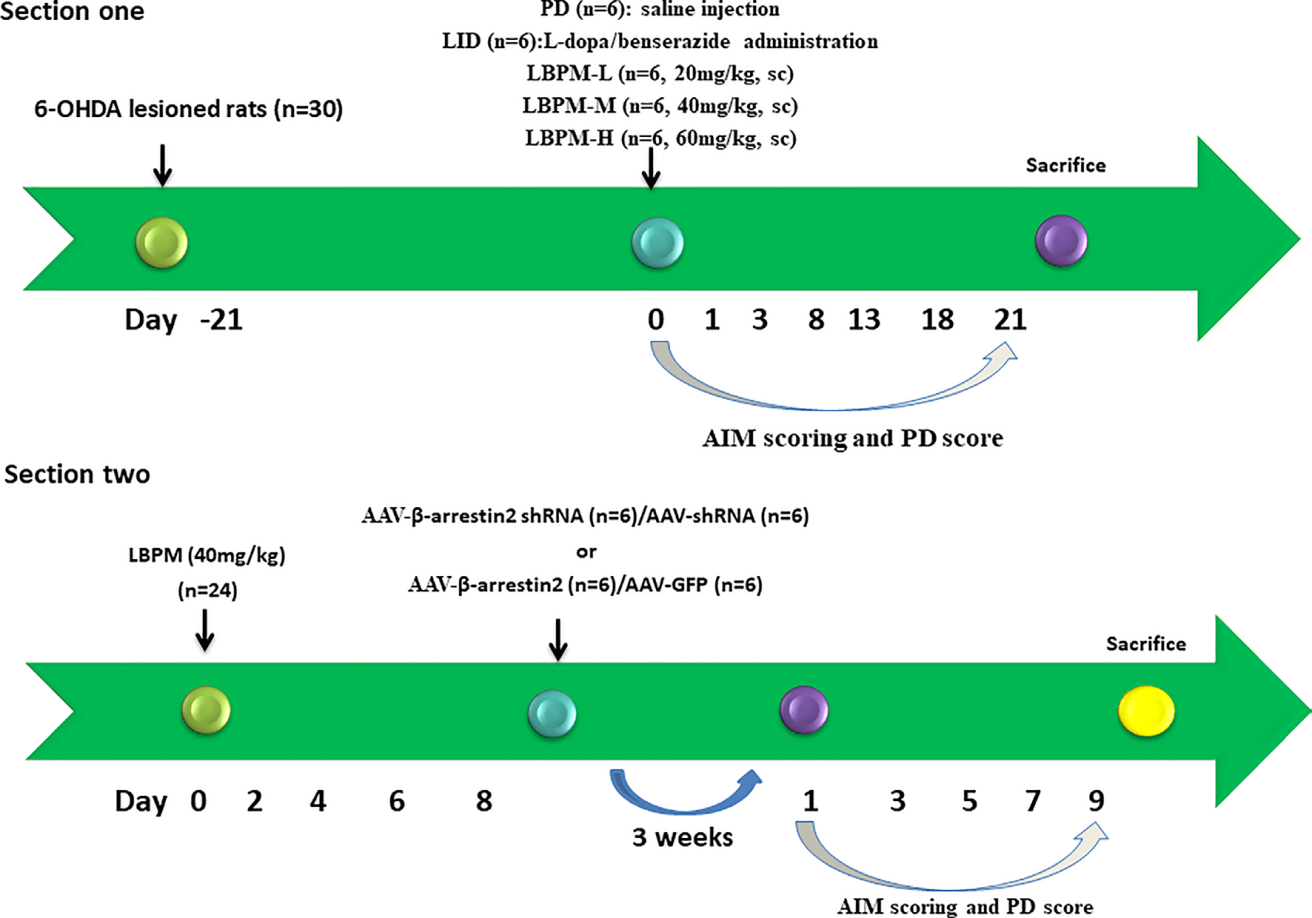
Schematic view of the experimental design. Section 1: SD rats (n = 30) were unilaterally injected with 6-OHDA in the MFB. Contralateral turning after apomorphine injection was tested 21 days later. Rats in the LID group (n = 6) were administrated once daily (9:00 am) with L-dopa (25 mg/kg, i.p.) plus benserazide (6.25 mg/kg, i.p.) for 3 weeks. PD rats in the microsphere groups received LBPM one time per week for 3 weeks and were further divided into three groups: LBPM-L [n = 6, 20 mg/kg, subcutaneous injection (sc)], LBPM-M (n = 6, 40 mg/kg, sc) and LBPM-H (n = 6, 60 mg/kg, sc). Section 2: PD rats (n = 24) received LBPM-M for one week, and then AAV-β-arrestin2 (β-arrestin2^+/+^) or AAV-β-arrestin2-shRNA (β-arrestin2^-/-^) or AAV-empty were injected into the striatum.

We have reported that the mean size of the LBPM nanoparticle was roughly 500 nm and the surface was smooth (Yang et al., 2012a, Yang et al., 2012b). After freeze-drying, the LBPM turned into well-dispersed powder (**Figure 1A and B**). **Figure 1C** shows that the two drugs (L-dopa/benserazide) sustained release within approximately 2 weeks (nearly 100%), demonstrating the effect of continuous dopamine stimulation. **Figure 2** describes the experimental design including section 1 and section 2.

**Figure 3:**
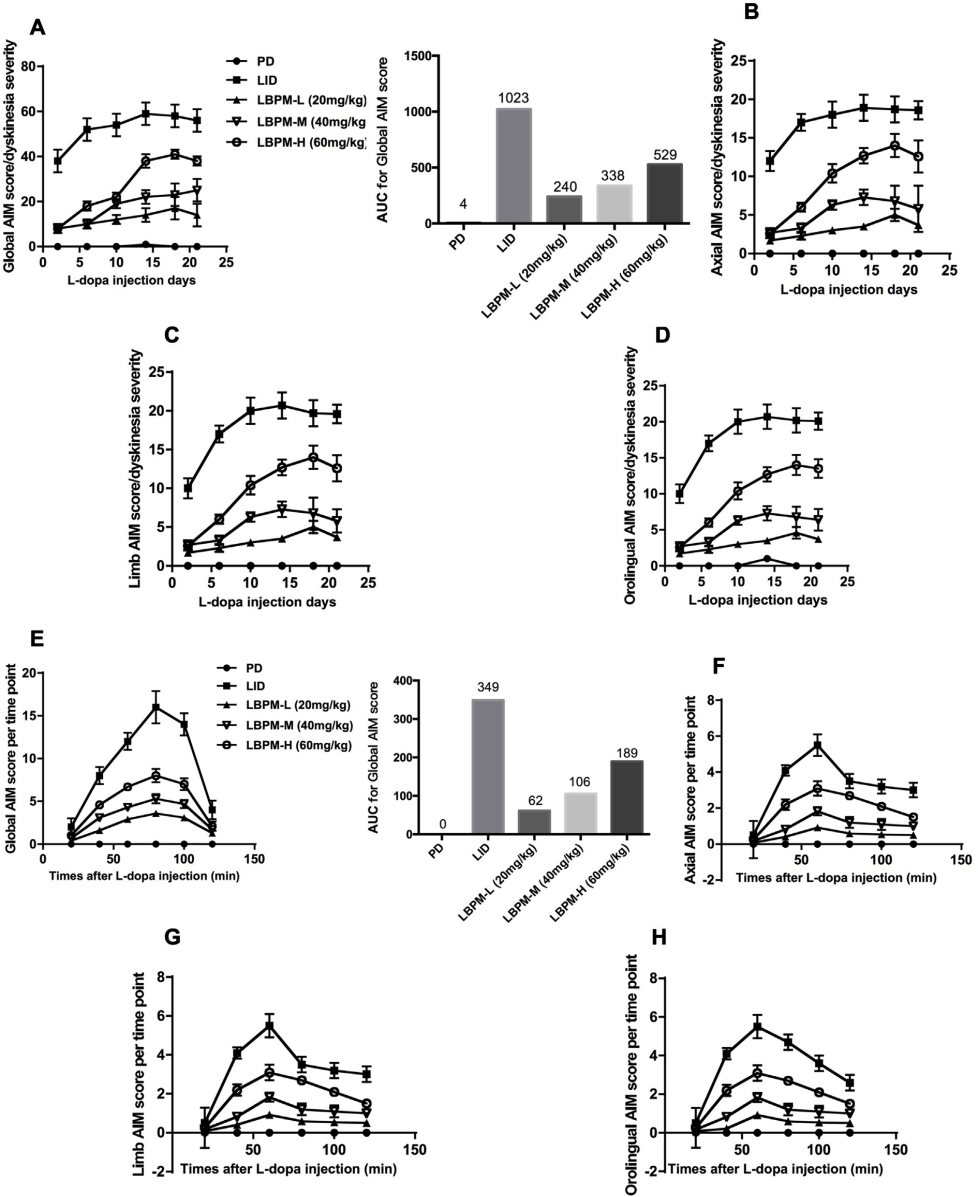
Time course of AIM score development in 6-OHDA-lesioned rats during a 3-week treatment with standard L-dopa, LBPM-L (20 mg/kg), LBPM-M (40 mg/kg) and LBPM-H (60 mg/kg). **(A, E)** Total AIM score and AUC for different groups; **(B, F)** axial AIM score; **(C, G)** limb AIM score and **(D, H)** orolingual AIM score. Thus, we found that LBPM prevented the development of LID in a 6-OHDA-lesioned rat model of PD. Data are presented as mean ± standard error of the mean; ^##^p < 0.001, ^#^p < 0.05 (n = 6 for each group, two-way repeated measures ANOVA followed by Bonferroni multiple comparison *post hoc* tests).

We used a well-established parkinsonian rat model by unilateral injection of the neurotoxin 6-OHDA into the right MFB. PD rats treated with L-dopa for 21 days developed a gradual increase in LID performance in terms of ALO AIM (*F_5,210_* = 14.06, p < 0.00001, **Figure 3A**). We found the mean ALO AIM ranged from 37.2 ± 4.6 on day 2 to 56.8 ± 4.28 on day 21 in LID rats (n = 6, **Figure 3A**). The same trend is likely to exist in axial AIM (**Figure 3B**), limb AIM (**Figure 3C**), as well as orolingual AIM (**Figure 3D**). On the contrary, PD rats cured with LBPM did not develop obvious LID within the 3-week period, which significantly differed from the LID groups in all testing sessions (*F_4,210_* = 234.0, p < 0.001), revealing a significant effect of duration and treatment. There was also significant interaction between the duration of L-dopa and LBPM treatment (*F_20,210_* = 2.532, p < 0.001). With regards to the LBPM-L (20 mg/kg) and LBPM-M (40 mg/kg) groups, median ALO AIM increased from 8.3 to 14.9 and 8.3 to 24.9, respectively, which represents an evident reduction compared with the LID group at the same time point (n = 6, P < 0.05 vs LID, **Figure 3A**). Moreover, the AUC was 240 in the LBPM-L group and 338 in the LBPM-M group (**Figure 3A**). Regrettably, LBPM-H (60 mg/kg, sc) rats also showed certain reduction in the ALO AIM relative to the rats receiving regular L-dopa, but the rats still displayed a mild dyskinesia after 2 weeks’ treatment, indicating that the content of L-dopa in the LBPM-H rats was enough to reach the dyskinesia performances. The reduction of total ALO AIM in the LBPM groups was more evident in the LBPM-L and LBPM-M groups rather than the LBPM-H group. When considering specific LID parameters, both peak and total ALO AIM showed significant reduction by 46% and 60% in the LBPM-M group compared with the LID group (n = 6, *F_20,210_* = 4.088, P < 0.0001 vs LID for treatment and time interaction, **Figure 3E**). The AUC was 62 in the LBPM-L group, 106 in the LBPM-M group and 189 in LBPM-H group (**Figure 3E**). In terms of dyskinesia duration, LID duration was 115 ± 12 min, and LBPM ranged between 102 ± 10, 104 ± 9, and 108 ± 11 min with various doses, none being significantly different from the LID group (P > 0.05 vs LID, **Figure 3E**). Similarly, there was a similar tendency in axial AIM (**Figure 3F**), limb AIM (**Figure 3G**), and orolingual AIM (**Figure 3H**).

### Effects of LBPM on Motor Responses

**Figure 4:**
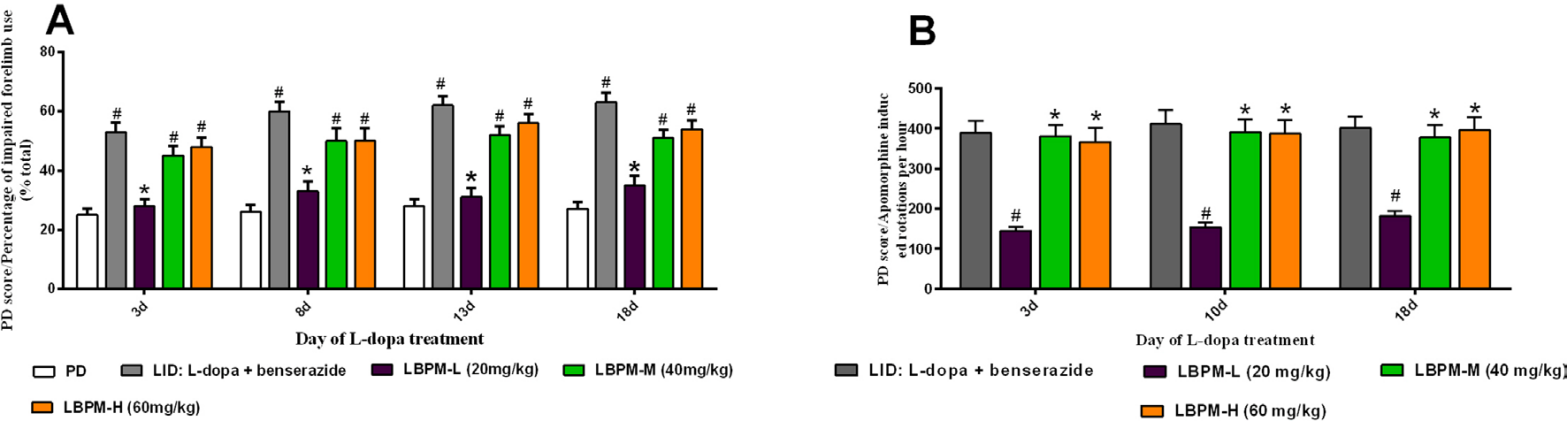
Effects of LBPM on motor responses. Animals were injected with either saline, standard L-dopa, LBPM-L (20 mg/kg), LBPM-M (40 mg/kg), or LBPM-H (60 mg/kg). **(A)** Forelimb functional test (FFT) and **(B)** apomorphine-induced rotation. The improvement in motor performance was similar in the LBPM-M compared with standard L-dopa administration. Data are presented as mean ± standard error of the mean; ^#^p < 0.05, *P > 0.05 (n = 6 for each group, two-way repeated measures ANOVA followed by Bonferroni multiple comparison *post hoc* tests).

We further sought to confirm whether the phenomenon of LBPM lowered the LID score by counteracting the therapeutic response to L-dopa. As depicted in **Figure 4**, FFT score was obviously reduced by L-dopa administration. L-dopa treatment increased the number of lesioned forelimbs utilized versus those at baseline on days 3, 8, 13, and 18 (n = 6, *F_3,620_* = 4.329, P = 0.0049, **Figure 4A**). Meanwhile, the improvement in motor performance was analogical in the LBPM-M or LBPM-H group compared with L-dopa treatment. There was no significant difference between the LBPM-M and LBPM-H groups, namely, LBPM-H did not produce an additional motor benefit over the LBPM-M administration. We speculated the potential reasons behind this paradoxical phenomenon in part since severe dyskinesia rats in the LBPM-H group may influence the FFT score. In addition, the number of rats in each group was small and needs to be expanded to validate this result. Furthermore, we found no significant reduction in contralateral rotations by apomorphine in the LBPM-M groups compared with the LID group (n = 6, *F_2,372_* = 0.5323, P = 0.5877 > 0.05, **Figure 4B**). In brief, rats in the LBPM-H group displayed mild to moderate dyskinesia features and no additional motor improvement in the LBPM-M group.

### Treatment with LBPM Increased the Level of β-Arrestin2

**Figure 5:**
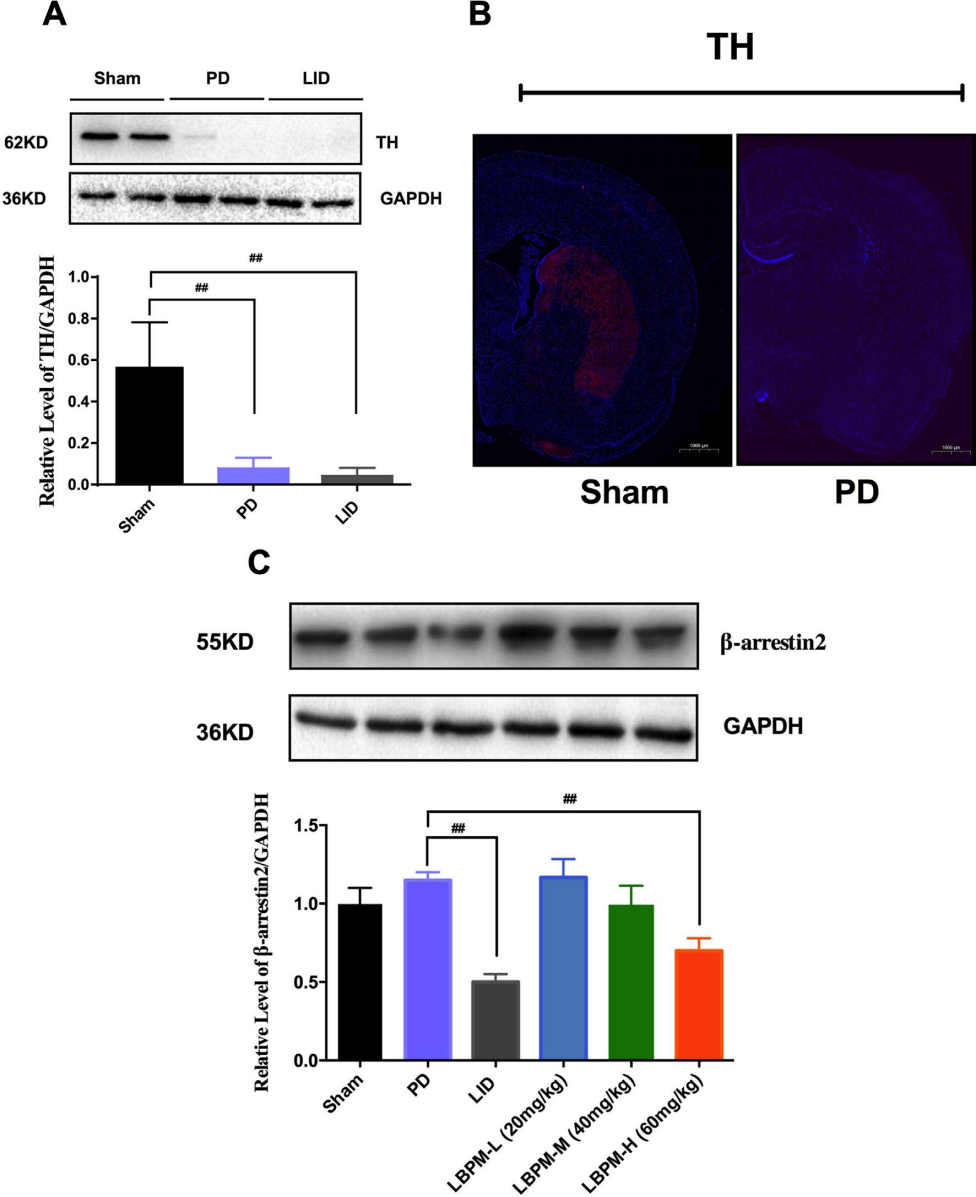
Treatment with LBPM increased the level of β-arrestin2 in the LID dorsal striatum. **(A)** Tyrosine hydroxylase (TH) protein level was dramatically decreased by nearly 80%–90% in the PD or LID rats when compared with the sham group. **(B)** Fluorescence slice of coronal plane from the striatum of the rat sham group or PD group stained with anti-tyrosine hydroxylase (TH). Scale bar represents 100 µm. **(C)** β-arrestin2 expression relative to actin level in each group. Data are presented as mean ± standard error of the mean; ^##^p < 0.001, *P > 0.05 (n = 6, ANOVA followed by LSD *post hoc* comparisons).

Tyrosine hydroxylase (TH) protein level extract from total striatum and positive neurons in the ipsilateral lesioned striatum were dramatically decreased by nearly 80%–90% in the PD or LID rats compared to the sham group (p < 0.001, **Figure 5A and B**). No significant changes were observed in TH levels between PD and LID rats (P > 0.05 vs PD, **Figure 5A and B**). There was an apparent decrease in the level of β-arrestin2 in the LID rats compared with PD rats (p < 0.001, **Figure 5C**). This reduction in β-arrestin2 expression did not occur in animals treated with LBPM-L or LBPM-M except for LBPM-H. Namely, LBPM-L or LBPM-M administration did not influence the level of β-arrestin2 and there was significant difference compared with LID (p < 0.001, **Figure 5C**). Meanwhile, there was no statistical discrepancy between LBPM-L and LBPM-M. Based on the previous results, we established that LBPM-M was the most effective and rational dose to prevent the LID performance and manage the PD motor symptoms.

### The Anti-Dyskinetic Effect of LBPM Was Overcome by the Ablation of β-Arrestin2 Expression

**Figure 6:**
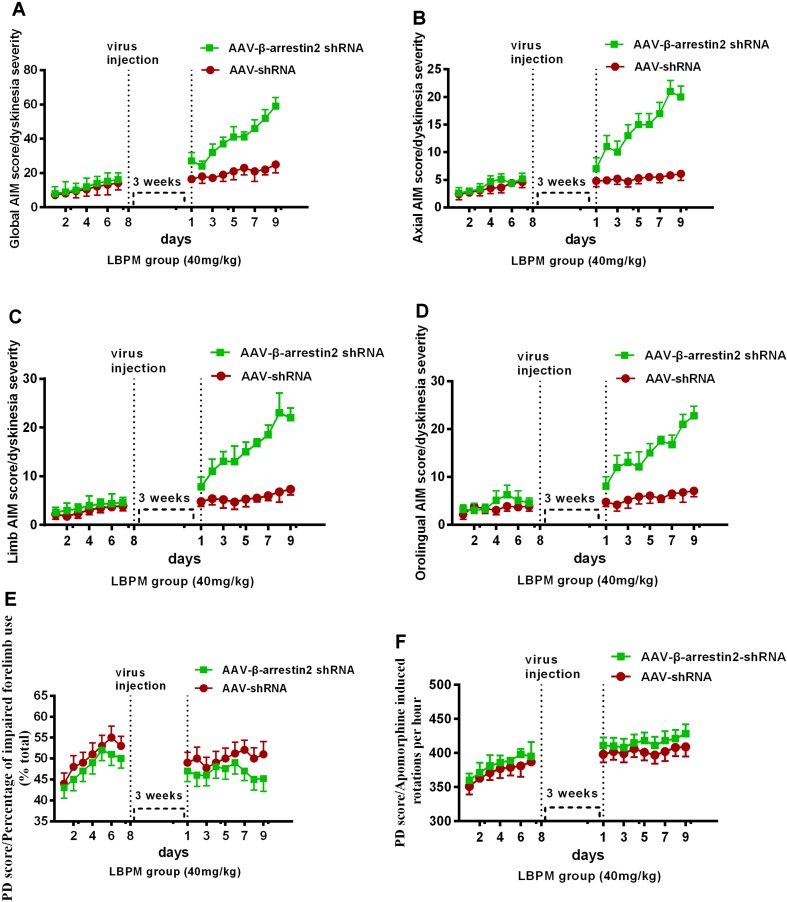
The anti-dyskinetic effect of LBPM was overcome by the ablation of β-arrestin2 expression. LBPM (40 mg/kg) induced a mild AIM score in PD rats for 7 days before virus injection. After virus injection, we found that genetic deletion of β-arrestin2 (AAV-β-arrestin2 shRNA) significantly enhanced the dyskinesia-like effects of LBPM treatment in terms of ALO AIMs **(A)**,** (B)** axial AIM score, **(C)** limb AIM score, and **(D)** orolingual AIM score. There was no difference between the AAV-β-arrestin2 shRNA and AAV-shRNA groups in terms of FFT score **(E)** and apomorphine-induced rotation **(F)**. ^##^p < 0.001, *P > 0.05 (n = 6, two-way repeated measures ANOVA followed by Bonferroni multiple comparison *post hoc* tests).

To test whether β-arrestin2 has a role in the anti-dyskinetic effect of LBPM, we generated AAV vectors encoding for either a short hairpin RNA (shRNA) to block β-arrestin2 expression (β-arrestin2^-/-^, AAV-β-arrestin2 shRNA) or a shRNA against firefly luciferase (AAV-shRNA) as a negative control. Before AAV injection, PD rats treated with LBPM (40 mg/kg) did not develop mild dyskinesia performance within a 1-week period. We then showed that genetic deletion of β-arrestin2 (AAV-β-arrestin2 shRNA) significantly enhanced the dyskinesia-like effects of LBPM treatment in terms of ALO AIMs (*F_8,126_* = 5.166, p < 0.001 for duration, *F_1,126_* = 90.82, p < 0.001 for treatment, **Figure 6A**) but not in the AAV-shRNA group. Analogously, we found this seemed to be the similar tendency in axial AIM (**Figure 6B**), limb AIM (**Figure 6C**), and orolingual AIM (**Figure 6D**). Meanwhile, there was no significant difference between the AAV-β-arrestin2 shRNA and AAV-shRNA groups in terms of FFT score (*F_8,198_* = 0.1327, p = 0.9977 for duration, *F_1,198_* = 3.225, p = 0.0740 for treatment, **Figure 6E**) and apomorphine-induced rotation (*F_8,318_* = 0.1488, p = 0.9967 for duration, *F_1,318_* = 2.208, p = 0.1383 for treatment, **Figure 6F**), suggesting AAV-β-arrestin2 shRNA has no motor effects. Taken together, these results showed that LBPM prevented LID maybe *via* β-arrestin2 in 6-OHDA lesioned PD rats.

### The Effects of LBPM Were Further Relieved by β-Arrestin2 Overexpression

**Figure 7:**
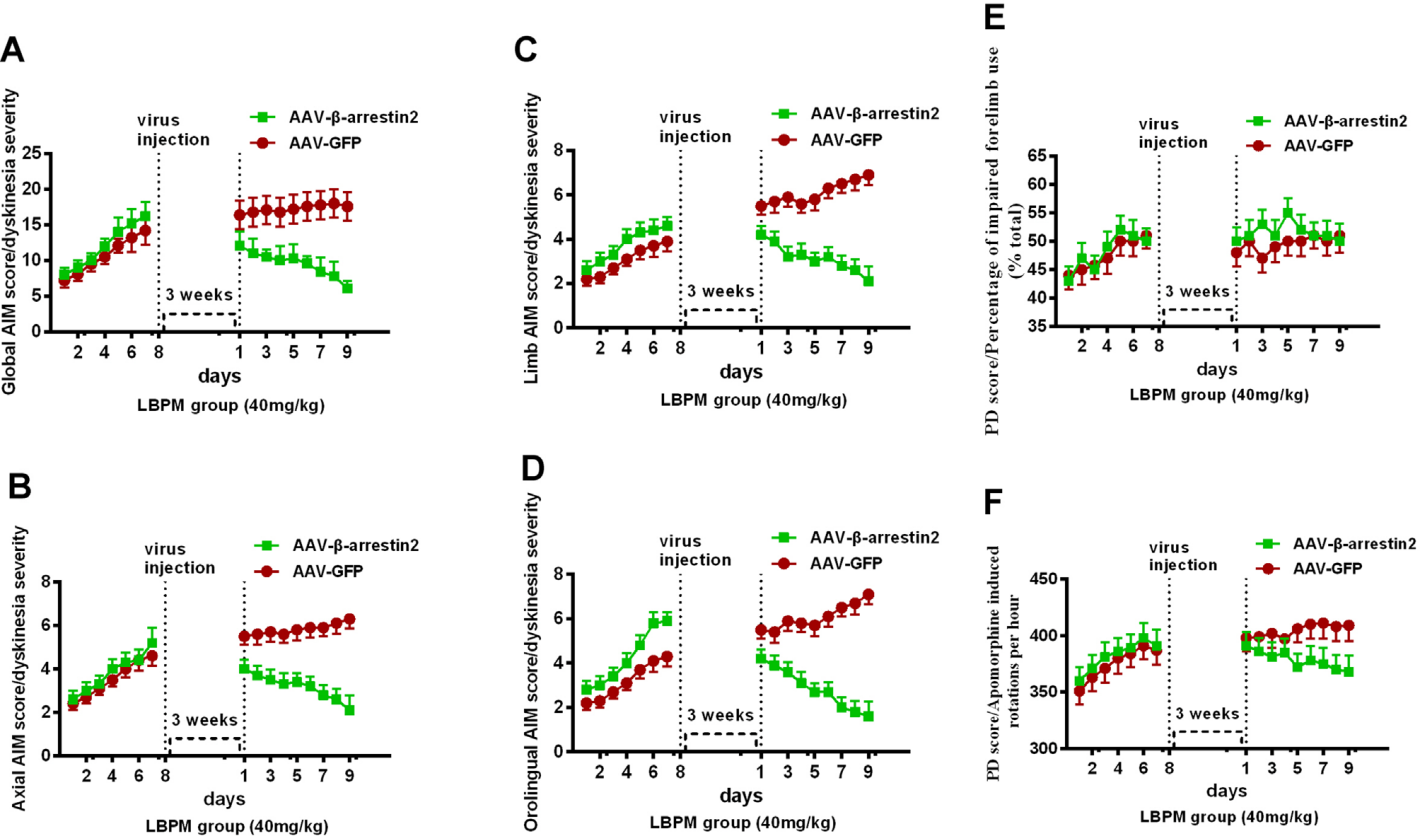
The effects of LBPM were further relieved by β-arrestin2 overexpression. LBPM (40 mg/kg) induced a mild AIM score in PD rats for 7 days before virus injection. After virus injection, we found that overexpression of β-arrestin2 (AAV-β-arrestin2) significantly further decreased ALO AIMs compared with AAV-GFP rats over 9 days **(A)**; **(B)** axial AIM score; **(C)** limb AIM score and **(D)** orolingual AIM score. There was no difference between the AAV-β-arrestin2 and AAV-GFP groups in terms of FFT score **(E)** or apomorphine-induced rotation **(F)**. ^#^p < 0.05, *P > 0.05 (n = 6, two-way repeated measures ANOVA followed by Bonferroni multiple comparison *post hoc* tests).

We constructed AAV encoding GFP (control) or rat β-arrestin2 tagged with GFP to detect easily overexpression of β-arrestin2 (β-arrestin2^+/+^, AAV-β-arrestin2). Intrastriatal administration of AAV-β-arrestin2 apparently decreased ALO AIMs compared to AAV-GFP rats within 9 days (*F_1,126_* = 76.67, p < 0. 0001, **Figure 7A**). Similarly, it is likely to be the same trend in axial AIM (**Figure 7B**), limb AIM (**Figure 7C**), and orolingual AIM (**Figure 7D**). Parkinsonian disability scores in both the FFT score (p > 0. 05, **Figure 7E**) and apomorphine-induced rotation (p > 0. 05, **Figure 7F**) were indistinguishable between observations made before and 3 weeks after the intrastriatal delivery of AAV-β-arrestin2.

## Discussion

L-dopa has offered revolutionary and miraculous benefits for averting disability and improving quality of life for PD patients. However, the occurrence of LID is a severe problem for PD patients. We have published before that LBPM is helpful in reducing the expression of LID in PD rats (Xie et al., 2014a), which was consistent with our present study. Meanwhile, it was found that treatment with LBPM increased the level of β-arrestin2 and led to dose dependent improvement in motor function by FFT and apomorphine-induced rotation. The results showed that the anti-dyskinetic effect of LBPM was reversed by selective deletion of β-arrestin2 in striatum neurons, and further relieved by β-arrestin2 overexpression. These data indicated that LBPM prevented the LID possibly *via* β-arrestin2 in PD rats.

Several studies have reported that pulsatile and intermittent treatment of L-dopa is attributed to the occurrence of LID (Xie et al., 2014b). Under normal physiological conditions, striatal dopamine liberate is relatively stable. Treatment with short half-life medicine, such as L-dopa, is connected with fluctuating plasma levels of the drug and subsequent oscillations in synaptic dopamine concentrations (Abbruzzese et al., 2012). Over time, this changeability is supposed to induce maladaptive changes in basal ganglia motor circuits, resulting in LID development (Wang and Zhang, 2016). Therefore, continuous delivery of L-dopa is helpful in reducing the emergence of the LID phenomenon. Warren et al. reported advanced PD subjects who received L-dopa-carbidopa intestinal gel had reduced off-time by 1.91 h and had improved activities of daily living and quality of life scores (Olanow et al., 2014). Recently, Wan et al. (2017) showed that continuous dopaminergic delivery (CDD) effectively avoided the overexpression of the D1R/Shp-2 signaling pathway, resulting in the reduction of LID performance in PD rats. In this paper, LBPM *via* microsphere technology was able to achieve sustained-release L-dopa/Benserazide for 2 weeks and realized CDD. Simultaneously, our results showed that PD rats treated with LBPM (40 mg/kg) did not develop obvious LID over the 3-week treatment period in terms of ALO AIMs, which was consistent with the previous studies of our group (Yang et al., 2012a; Yang et al., 2012b; Xie et al., 2014a). Then we focused on the molecular mechanism of LBPM in LID to clarify the association of LBPM and LID. Indeed, the detailed mechanisms behind differences between pulsatile and continuous L-dopa (LBPM) are not absolutely clear, but it is considered that intermittent L-dopa induces pulsating activation of DRs, especially D1R in the medium spiny neurons (MSNs) of the striatum (Feyder et al., 2011). The pathological enhancement in the number of D1R by L-dopa administration is likely to result in LID and denotes a potential target for therapy (Mosharov et al., 2015). Darmopil et al. showed that D1R inactivation abolished LID scores and associated molecular changes. Inactivation of the D2R had no obvious effect on the behavioral or molecular response to L-dopa (Darmopil et al., 2009). In line with this theory, recent research has shown that LID is reduced by promoting GPCR desensitization (Nishi and Shuto, 2017). In addition, strengthening β-arrestin2 activity seems to be a suitable strategy because β-arrestin2 is supposed to desensitize receptor signaling (Ismail et al., 2015). In the present study, we found that there was a mild decrease in the level of β-arrestin2 in the LID rats by L-dopa pulsatile administration compared with PD rats. This reduction in β-arrestin2 expression did not occur in animals treated with LBPM.

β-Arrestin2, as a non-visual arrestin, can transduce GPCR signals by forming protein complexes to mediate GPCR desensitization, degradation and recycling (Zhan et al., 2011). Urs et al. (2015) reported that β-arrestin2 overexpression significantly reduced LID while maintaining the therapeutic effect of L-dopa in knock-out PD mice. Our present results are in line with previous research showing that the effects of LBPM were further relieved by β-arrestin2 overexpression. On the contrary, genetic deletion of β-arrestin2 (AAV-β-arrestin2 shRNA) significantly deteriorated the dyskinesia-like effects of LBPM treatment in terms of ALO AIMs but not in the AAV-shRNA group. Taken together, all these data indicated that β-arrestin2 played a pivotal role in behavioral sensitization after L-dopa treatment.

In conclusion, our work opens the way for a possible indication of CDD therapy in PD, namely LBPM can “rebalance” the response of striatal neurons to L-dopa, thus preventing troublesome side effects without affecting motor efﬁcacy. In short, our experiments provided evidence that LBPM prevention of LID behavior was likely through β-arrestin2, suggesting that a therapy modulating β-arrestin2 may provide a more effective anti-dyskinetic approach with a low risk of untoward effects.

## Ethics Statement

The study was approved by the Ethics Committee of the First Affiliated Hospital of Wenzhou Medical University. In addition, the whole animal experiments carried out on the basis of the Guide for the Care and Use of Laboratory Animals issued by the Institute of Laboratory Animal Resources at the Commission on Life Sciences of the National Research Council and published by National Academy Press.

## Author Contributions

W-WW, X-RZ, and Z-RZ made substantial contributions to conception and design. ZW, S-YC, and J-YL re-analyzed all the data and interpreted the data. C-LX was involved in drafting the manuscript. All authors read and approved the final manuscript.

## Funding

The study was supported by the Projects of National Science Foundation of China (No. 81600977) and the Projects of Wenzhou City Committee of Science and Technology (Y20180137 and Y20170067) and the Projects of Natural Science Foundation of Zhejiang Province (Y19H090059).

## Conflict of Interests Statement

The authors declare that the research was conducted in the absence of any commercial or financial relationships that could be construed as a potential conflict of interest.
